# Detection of Vesicular Stomatitis Virus Indiana from Insects Collected during the 2020 Outbreak in Kansas, USA

**DOI:** 10.3390/pathogens10091126

**Published:** 2021-09-02

**Authors:** Bethany L. McGregor, Paula Rozo-Lopez, Travis M. Davis, Barbara S. Drolet

**Affiliations:** 1 Arthropod-Borne Animal Diseases Research Unit, Center for Grain and Animal Health Research, Agricultural Research Service, United States Department of Agriculture, Manhattan, KS 66502, USA; Bethany.McGregor@usda.gov (B.L.M.); Travis.Davis2@usda.gov (T.M.D.); 2 Department of Entomology, Kansas State University, Manhattan, KS 66506, USA; paularozo@ksu.edu

**Keywords:** vesicular stomatitis virus, Indiana, VSV-IN, *Rhabdoviridae*, Vesiculovirus, *Culicoides*, *Simulium*

## Abstract

Vesicular stomatitis (VS) is a reportable viral disease which affects horses, cattle, and pigs in the Americas. Outbreaks of vesicular stomatitis virus New Jersey serotype (VSV-NJ) in the United States typically occur on a 5–10-year cycle, usually affecting western and southwestern states. In 2019–2020, an outbreak of VSV Indiana serotype (VSV-IN) extended eastward into the states of Kansas and Missouri for the first time in several decades, leading to 101 confirmed premises in Kansas and 37 confirmed premises in Missouri. In order to investigate which vector species contributed to the outbreak in Kansas, we conducted insect surveillance at two farms that experienced confirmed VSV-positive cases, one each in Riley County and Franklin County. Centers for Disease Control and Prevention miniature light traps were used to collect biting flies on the premises. Two genera of known VSV vectors, *Culicoides* biting midges and *Simulium* black flies, were identified to species, pooled by species, sex, reproductive status, and collection site, and tested for the presence of VSV-IN RNA by RT-qPCR. In total, eight positive pools were detected from *Culicoides sonorensis* (1), *Culicoides stellifer* (3), *Culicoides variipennis* (1), and *Simulium meridionale* (3). The *C. sonorensis*- and *C. variipennis*-positive pools were from nulliparous individuals, possibly indicating transovarial or venereal transmission as the source of virus. This is the first report of VSV-IN in field caught *C. stellifer* and the first report of either serotype in *S. meridionale* near outbreak premises. These results improve our understanding of the role midges and black flies play in VSV epidemiology in the United States and broadens the scope of vector species for targeted surveillance and control.

## 1. Introduction

Vesicular stomatitis virus (VSV; family *Rhabdoviridae*, genus *Vesiculovirus*) is the causative agent of vesicular stomatitis (VS) disease in horses, cattle, and pigs in the Americas. Two serotypes have been described, VSV New Jersey (VSV-NJ) and Indiana (VSV-IN). In the United States (U.S.), the disease primarily affects horses and cattle, producing lesions and ulceration of the mouth, teats, and coronary bands of affected animals. While infection does not typically result in mortality in any of the species, clinical disease in cattle and swine closely resembles foot and mouth disease. Therefore, VSV is a reportable disease that results in quarantines and animal movement restrictions on premises with positive animals [1,2]. Due to these restrictions and effects of the virus itself, outbreaks of VSV on dairy and beef operations have resulted in significant economic impacts. These include estimated average financial losses of USD 15,565/beef cattle ranch in Colorado [3], approximately USD 50,000 in losses to a single dairy farm in Idaho [4], from USD 200 to USD 250/dairy cow affected with oral lesions in California [5] and Colorado [6], and up to USD 570/dairy cow for those with teat lesions [6].

VSV is maintained in an endemic transmission cycle in South and Central America. Sporadic outbreaks with the VSV-NJ serotype in the U.S. occur on a 5–10-year cycle, often with viral incursions into the southwestern states of Texas, Arizona, and New Mexico, and spreading northward into Colorado and Wyoming [7]. Outbreaks with the VSV-IN serotype in the U.S. are much less frequent with prior outbreaks only reported in 1966 and 1997–1998 [7,8]. Transmission can occur animal to animal by direct contact, through fomites contaminated with virus from shedding animals, and through mechanical and biological vector transmission [9]. The suspected biological vector range for this pathogen is extensive, encompassing black flies (*Simuliidae*), biting midges (*Ceratopogonidae*), mosquitoes (*Culicidae*), and sand flies (*Psychodidae* subfamily *Phlebotiminae*) [9]. Information on specific vector species and regional variation in vector communities is sparse. To date, confirmed competent North American vectors are from three genera of these biting Diptera: *Lutzomyia shannoni* [10], *Culicoides sonorensis* [11,12], *Simulium vittatum*, and *S. notatum* [13].

To estimate vector-borne VSV transmission risk to animals in any given region, it is critical to identify all competent vector species, and determine which species are present. These incriminated Dipteran vectors differ substantially in their individual ecologies, especially as it pertains to larval habitat requirements, which vary from fully aquatic to fully terrestrial. Black fly larvae are fully aquatic and require fast-flowing fresh water to complete their development, making them common in rivers and streams proximal to bloodmeal sources [14]. Biting midge larvae inhabit semi-aquatic habitats, typically at the water–soil interface of waterbodies such as ponds, streams, puddles, and springs [15]. Sand fly larvae require moist, organic terrestrial soil conditions without standing water. Therefore, the primary vector taxa present in an area, and the relative risk of VSV transmission, is often a byproduct of larval habitat availability. Adult dispersal, which also differs significantly between these taxa, can also assist with the movement of vectors and associated pathogens. Sand flies have characteristically weak flight ability and short dispersal of just a few hundred meters [16]. Biting midges are slightly stronger fliers with average dispersal distances of 1–2.5 km [17]. Finally, black flies are the strongest fliers of these three, with some species dispersing more than 100 km [18] and single recorded continuous flight distances of up to 5.25 km [19]. Due to their presence in running water, black fly larvae can also disperse both passively and actively by water flow [20] and both biting midges and black flies are suspected to move on air currents over long distances [21,22,23,24,25,26], potentially contributing to the movement of VSV over large geographic areas.

The latest U.S. VSV-IN outbreak started in 2019 affecting eight states (Colorado, Kansas, Nebraska, New Mexico, Oklahoma, Texas, Utah, and Wyoming) with a total of 1144 affected premises [27]. In 2020, a second year of the outbreak affected eight states (Arizona, Arkansas, Kansas, Missouri, Nebraska, New Mexico, Oklahoma, and Texas) with a total of 326 affected premises [28]. The 2020 outbreak expanded further eastward than in typical years, reaching farms as far east as Kansas and Missouri for the first time in decades. In total, 26 counties in Kansas reported VSV-positive premises, mainly within the southeastern portion of the state [29]. This expansion into an area representing unique vector communities provided an opportunity to further investigate the potential vector breadth of VSV-IN outside the southwest. We report on virus detection efforts conducted on field-caught *Culicoides* biting midges and *Simulium* black flies at two VSV-positive premises in eastern Kansas in 2020.

## 2. Results

### 2.1. Field Collections

**Figure 1:**
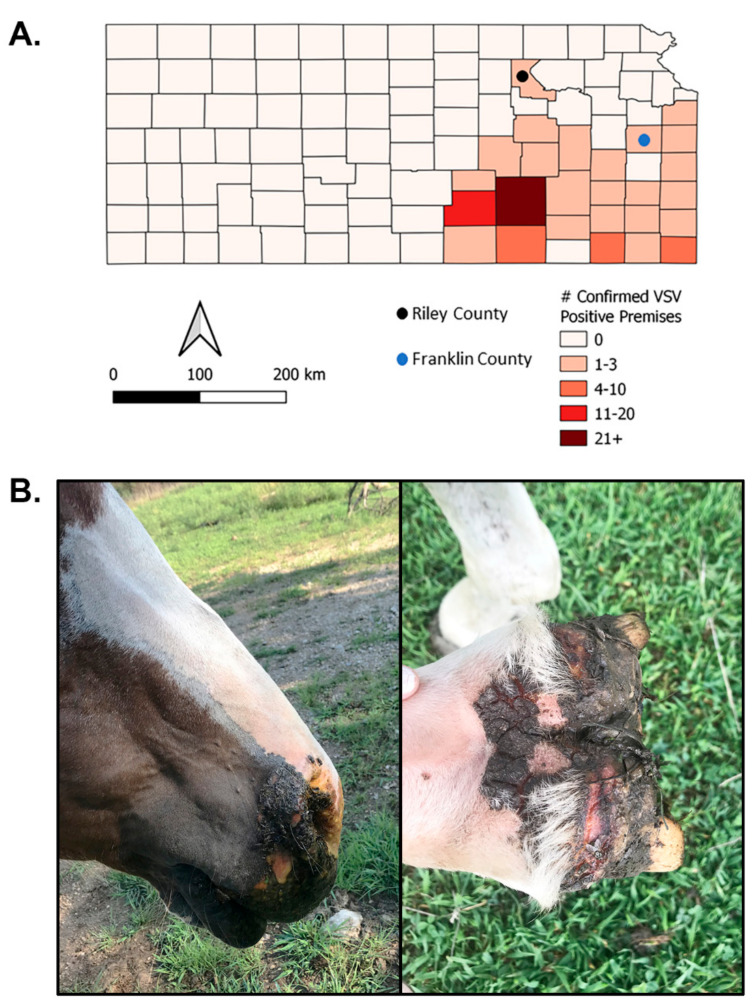
Insect collections on vesicular stomatitis virus-Indiana (VSV-IN)-positive premises in Kansas, USA. (**A**) Map depicting the number of USDA-APHIS confirmed VSV-IN cases per county. Insects were collected in Riley and Franklin counties (solid circles). (**B**) Clinically affected horse on Franklin County premises. Map generated using QGis with publicly available shapefiles and data. (Photographs used with permission from owner, 2021).

Collections were conducted on two farms in Kansas: one in Riley County and one in Franklin County (Figure 1). In total, 1396 biting insects were collected and sorted into 154 pools for VSV testing including 40 *Simulium* and 1356 *Culicoides* individuals. All *Simulium* were collected from the Franklin County site and identified as female *S. meridionale*. Eight species of *Culicoides* were identified and pooled separately by sex, reproductive status, and collection site (Table 1 and Table 2). Of the *Culicoides* collected, 469 were from Franklin County and 927 were from Riley County.

### 2.2. VSV Detection by RT-qPCR

**Figure 2:**
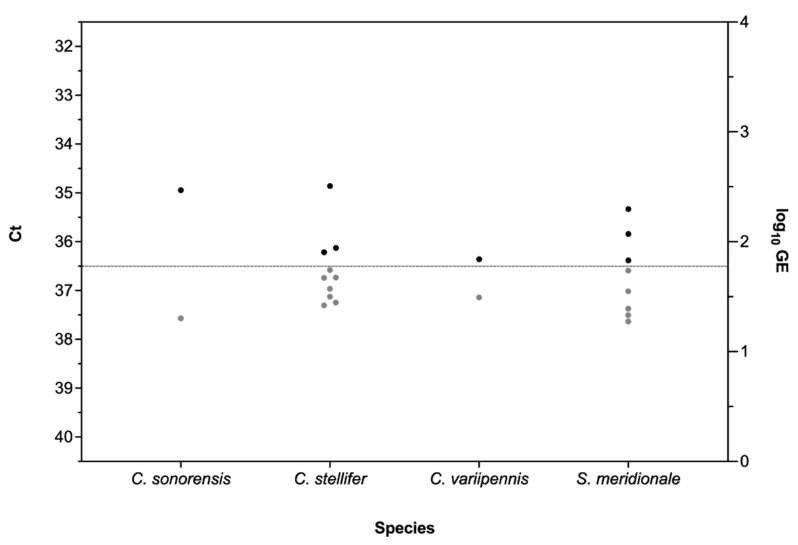
Detection of VSV-IN in field-collected insects from two counties in Kansas during the 2020 outbreak. Positive insect species (black dots) above RT-qPCR cycle threshold (Ct) cutoff point (≤36.5). Ct values (left Y-axis) and calculated log_10_ viral genome equivalents (right Y axis).

Of the 154 pools tested, RT-qPCR-positive results (Ct ≤ 36.5) were identified for eight pools (Figure 2), including three from Franklin County and five from Riley County. The positive Franklin County pools were all female *Simulium meridionale* (Ct = 35.84, 36.38, 35.33). The positive Riley County pools included three pools of gravid *C. stellifer* (Ct = 35.84, 36.38, 35.33), one pool of nulliparous *C. sonorensis* (Ct = 34.94), and one pool of nulliparous *C. variipennis* (Ct = 36.36).

### 2.3. Sequencing

Partial sequencing of the VSV hypervariable phosphoprotein (P) gene was successfully conducted on one pool of nulliparous *C. sonorensis*, producing a 337 nucleotide (nt) sequence (GenBank accession: MZ703188). As of July 12, 2021, only seven VSV-IN 2019–2020 outbreak sequences were publicly available (BLASTN 2.12.0; [30]). The *C. sonorensis* sequence most closely matched four near-complete genomes from the 2020 outbreak in Kansas (GenBank accessions: MW373776 to MW373779) and three genome sequences from the 2019 outbreak in the southwestern U.S. (GenBank accessions: MT437283 to MT437285) (92.6% identity, 100% query cover, 4e-140 E-value for all matches) (Supplemental Table S1). Additionally, 140 nt sequences were obtained for pooled samples of the Riley County *C. stellifer* (GenBank accession: MZ703186) and the Franklin County *S. meridionale* (GenBank accession: MZ703187). These sequences had 100% identity to each other and 95.68% identity to the available 2019/2020 sequences (99% query cover, 1e-61 E-value) (Supplemental Table S1). Unfortunately, whole genome sequencing attempts from positive pools were unsuccessful.

### 2.4. Field Infection Rates

Two field infection rate metrics were calculated to provide a measure of the comparative epidemiological contribution of the implicated vector species in this study. The metrics calculated include the minimum infection rate (MIR), a conservative estimate, and the maximum likelihood estimate (MLE), a more robust estimate of field infection rates. Both MIR and MLE were calculated for positive biting midge species at the Riley County site and expressed as total positive insects per 1000 in the population. The MIR for *C. stellifer* was 11.24 with an MLE of 12.25 (95% CI: 2.61–46.39). For *C. sonorensis*, the calculated MIR was 6.76 and MLE was 6.81 (95% CI: 0.40–33.61). The MIR for *C. variipennis* was 3.76 with an MLE of 3.66 (95% CI: 0.22–17.47). At the Franklin County site, the MIR for *S. meridionale* was 75. MLE could not be calculated for *S. meridionale* since the few pools tested did not allow the generation of robust estimates.

## 3. Discussion

This is the first report with data documenting putative vector species for VSV transmission during an active outbreak in eastern Kansas. While *C. sonorensis* has been confirmed as a competent vector [11,12], our finding of positive nulliparous *C. sonorensis* is novel. Nulliparous midges have never taken a blood meal, indicating that this positive pool is the result of an alternative infection pathway such as venereal or transovarial transmission. Venereal transmission has been documented in experimentally infected *C. sonorensis* [31] and the presence of VSV in developing oocytes and the ovarial epithelium of experimentally infected females suggest that transovarial transmission may also be possible [12]. Although few sequences are currently available in public databases for the 2019–2020 VSV-IN outbreak, analysis of sequencing data for this pool showed it was divergent from, but most closely associated with, 2019 bovine and 2020 horse isolates. Several factors may have contributed to the nulliparous pool having lower percent identity with the available mammalian isolate sequences than did parous pools that would have fed on infected animals. VSV is a non-segmented negative-stranded RNA virus with extremely wide host tropism and a relatively constant mutation rate of approximately 6 Log_10_ substitutions per nucleotide per round of replication in mammalian cells [32]. An insect-to-insect transmitted virus, with the genetic bottlenecks and selective pressures inherent to transovarial or venereal transmission, may be expected to show greater divergence from mammalian-derived (blood meal) virus populations. The paucity of publicly available 2019–2020 isolate sequences and our inability to obtain sequence from more than a single fragment of the hypervariable region of the P gene limited the robustness of our comparative analysis. However, the VSV RT-qPCR and confirmatory sequence results support our hypothesis of vertical or venereal VSV transmission occurring in the field-collected nulliparous *C. sonorensis* pool.

In addition to the *C. sonorensis* nulliparous pool, one pool of nulliparous *C. variipennis* was also RT-qPCR-positive. *C. variipennis* is closely related to *C. sonorensis*, having only been recognized as distinct species after a reassessment in 2000 [33]. Despite their close phylogenetic association, it is generally accepted that *C. sonorensis* is the vastly more competent vector species for most *Culicoides*-borne viruses [34,35] and the majority of vector competence studies focus on *C. sonorensis* due to availability of laboratory colonies. Our results indicate that at least some degree of vector competence for VSV is possible for *C. variipennis*, although infection rates for this species were low (3.66 MLE for *C. variipennis* compared to 6.81 for *C. sonorensis*) suggesting a low likelihood that this species is contributing substantially to transmission. Despite this, our finding of VSV-positive nulliparous *C. variipennis* emphasizes the need for additional vector competence studies involving *Culicoides* species other than *C. sonorensis*.

*Culicoides stellifer* has never formally been implicated as a vector for VSV, although VSV-NJ was isolated from a single pool of this species during an outbreak in Colorado [36]. This species is also a suspected vector for the orbiviruses bluetongue virus and epizootic hemorrhagic disease virus [37,38]. Moreover, *C. stellifer* has a broad geographic distribution throughout the U.S. [39], is often found in areas inhabited by grazing herbivores, and has documented blood-feeding events on horses [40,41,42,43]. In some parts of the U.S., *C. stellifer* often dominates collections of biting midges [44,45], leading to further speculation that even at low levels of competence for arboviruses, higher abundance could lead to an increase in overall vectorial capacity for this species [38,46]. Calculated infection rates for *C. stellifer* were high compared with the other positive *Culicoides* species in this study at 11.24 MIR and 12.25 MLE, suggesting that this species may play a significant role in VSV-IN transmission. However, because insects were collected in ethanol, virus isolations to confirm potential transmission were not possible.

Two black fly species, *S. vittatum* and *S. notatum*, have been implicated as competent vectors of VSV-IN in the U.S. [13]. For VSV-NJ, only *S. notatum* has been found to be competent in laboratory assays [47], while *S. bivittatum* has been implicated due to virus detections in field-collected specimens [48]. While *S. vittatum* is known to occur within the sampled region of Kansas, no individuals of this species were collected during this effort at either of the outbreak premises sampled. *Simulium meridionale* has not previously been implicated as a vector of either VSV serotype and historically was believed to feed primarily on birds [49]. This species is known to transmit the turkey parasite *Leucocytozoon smithi* [50], and large swarms have resulted in avian simuliotoxicosis [51]. However, additional evidence has shown that this species will feed on mammals, including humans and cattle [52,53]. This black fly species was the third most common biting insect sampled on the Franklin County farm at which the primary available hosts were horses. An exceptionally high MIR value (75) was calculated for this species, which could indicate a substantial potential for VSV-IN transmission. However, comparably low numbers of individuals of this species were available for testing, so this infection rate may be artificially high. Further studies to elucidate the vector competence of this *Simulium* species are warranted to better understand its potential role in the transmission of VSV-IN in the U.S. Because neither *C. stellifer* nor *S. meridionale* have been previously implicated as vectors, sequencing was attempted to confirm the positive RT-qPCR result. A hypervariable P gene sequence was obtained; however, the minimal length (140 nt) made sequence analysis possible but less robust than desired. In addition to alignments and comparisons with all available sequences, we sequenced our positive control strain to rule out potential plate contamination. Sequences from the two species pools, from two different counties over 100 miles apart, were 100% identical to each other and divergent from the plate control.

The vectors collected on these two farms reflect the availability of larval habitats for biting midges and black flies. The Franklin County farm has a flowing stream moving through the center of the property that is conducive to the development of black fly larvae. In addition to the stream, numerous low-lying areas and a pond were present on this property, creating conditions favorable for the breeding of several biting midge species. While black flies have been collected around Riley County [54], the lack of adequate flowing water resources near the VSV-positive premises and proliferation of low lying, semi-aquatic habitats led to great abundance of biting midges and the absence of black flies in our collections from this site. Black flies are capable fliers that can disperse over great distances. Thus, while the lack of great abundance of black flies was expected, the lack of any individuals in our collections was surprising. It is likely that the availability of prime larval habitat and hosts closer to their emergence sites within the county likely precluded black flies from dispersing to the VSV-positive premises. This result highlights the importance of considering the ecology of vector species when assessing which VSV vectors to target and predicting vector-borne disease transmission risk.

For both *C. stellifer* and *S. meridionale*, these first reports of VSV-IN positives fulfill two of the four criteria necessary for the implication of vector species [55]. Virus was detected in field-collected specimens of both species that lacked any visible blood during an active VSV-IN outbreak, which fulfills the first criterium. Our collections also fulfilled the fourth criterium by showing an association of the infected arthropods with affected vertebrate populations, although additional studies on host associations, especially for *S. meridionale*, would strengthen this criterium. The remaining two criteria require completion of laboratory vector competence studies showing the ability for these species to become infected and then biologically transmit the virus. Completion of these laboratory assays with these two species is greatly needed considering the high calculated infection rates for both during this study. At this time, these species can be categorized as suspected, although not confirmed, vectors of VSV-IN until the completion of all criteria.

No positive samples were recovered from other midge species collected, including *C. haematopotus* and *C. crepuscularis*, both species that have been implicated as suspected vectors of other arboviruses [56]. Despite testing 65 pools from these two species, the lack of positives gives us confidence that these species were not likely contributing to the transmission of VSV-IN on the study premises. However, it is also possible that conducting collections solely with blacklight LED traps could have biased against the collection of some species that are attracted to other light wavelengths [57,58], or even against infected vectors, which has been proposed for *C. sonorensis* infected with BTV [59].

In this study, insect collections were performed within two weeks of VSV lesion onset dates. No additional cases were reported for the locations and no additional collections were made. Therefore, it is unclear how long infected insects persisted on these premises and whether persistent infection in midge populations could have the potential for overwintering. A study evaluating the phylogenetics of isolates associated with VSV-NJ outbreaks in the U.S. in 2005 and 2006 found that the 2006 outbreak was the result of overwintering rather than a novel introduction event [60] with *S. bivittatum* involved at the start of the outbreak [48]. An overwintering event is especially concerning considering the potential transovarial or venereal transmission suspected due to VSV-IN detection in nulliparous *C. sonorensis* and *C. variipennis*. Based on laboratory studies, both transovarial or venereal transmission are speculated maintenance mechanisms for VSV in Phlebotomine sandflies [61] and *C. sonorensis* [31]. As of August 24, 2021, no VSV-IN cases have been documented within the U.S. in 2021. Although *Culicoides* are well-adapted to survive cold temperatures, a particularly hard freeze from a polar vortex in February 2021 may have impacted the survival of overwintering biting midges. It is unclear how venereally or vertically transmitted viral infections could be affected by this particularly extreme weather event.

In conclusion, we have identified four species of biting Diptera that were positive for VSV-IN during the 2020 expansion into Kansas: *C. sonorensis*, *C. stellifer*, *C. variipennis*, and *S. meridionale*. Calculated infection rates for these species were variable, with the greatest rates calculated in *S. meridionale* and *C. stellifer* and the lowest rates in *C. variipennis*. Each of these species represents unique biology and ecology that must be considered when optimizing control and surveillance strategies moving forward. This is also the first field detection of VSV-IN-positive nulliparous individuals indicating transovarial or venereal transmission outside of a laboratory environment. These data contribute substantially to our knowledge of the epidemiology of VSV-IN in the U.S. and will improve our ability to conduct targeted surveillance and control of vector species in the event of continued outbreaks.

## 4. Materials and Methods

### 4.1. Field Collections

Insects were collected during the 2020 outbreak at two sites in Kansas where confirmed VSV-IN-positive horses were present. The premises in Riley County reported one equine case (of 14 horses on site) and the Franklin County premises had three equine cases (of 7 horses on site) (Figure 1). At both sites, Centers for Disease Control and Prevention (CDC) miniature light traps baited with black light LED arrays (BioQuip Inc., Rancho Dominguez, CA, USA) were used to collect insects. When available, CO_2_ (dry ice) was used to improve trap efficacy. Collections took place overnight on July 30 and August 5–6 on the Franklin County site (lesion onset date was July 23) [62,63] using five traps per night and July 31 on the Riley County site (estimated lesion onset date July 20) [63,64] using seven traps. Insects were collected directly into 95% ethanol. Traps were recovered the following morning and collection tubes were immediately returned to the lab and placed into a freezer at −80 °C prior to processing.

### 4.2. Species Identification and Pooling

All insect sorting and identification was conducted on a chill table (BioQuip Inc.). Midges were identified to species and sex using morphological keys [15,33] and further identified as to physiological status (parous, nulliparous, gravid) using abdominal cuticular pigmentation [65,66]. Black flies were also identified to species and sex [14]. Insects were pooled in 2 mL microcentrifuge tubes containing 500 µL TRIzol reagent according to species, physiological status (for *Culicoides* only), site, and date. *Culicoides* were pooled in groups of ≤20 individuals and *Simulium* in pools of ≤5 individuals. Nulliparous, blood fed, and male midges were all pooled separately. Gravid and parous midges were combined in some pools.

### 4.3. RNA Extraction and RT-qPCR for VSV-IN Detection 

Frozen pools of midges and black flies stored in 500 μL of TRIzol were thawed on ice, two 2.4 mm stainless steel beads (Omni Inc., Kennesaw, GA, USA) were added, and tubes were homogenized by shaking at 3.1 m/s with a Bead Mill Homogenizer (Omni Inc.). Additional 500 μL of TRIzol were added for a final volume of 1000 μL. Samples were centrifuged at 12,000 × g for 6 min to pellet debris. Total RNA was extracted using Trizol-BCP (1-Bromo-3-chloropropane; ThermoFisher Life Technologies, Inc. Waltham, MA, USA). RNA was precipitated using isopropanol, washed in 75% ethanol, and eluted in 40 μL of nuclease-free water. RNA extracts were analyzed using TaqMan Fast Virus 1-Step MasterMix (Applied Biosystems; Thermo Fisher Scientific, Inc.) in a RT-qPCR targeting the L segment [67]: forward primer VSVIN+7230: 5′-TGATACAGTACAATTATTTTTGGGAC-3; reverse primer VSVIN-7456: 5′-GAGACTTTCTGTTACGGGATCTGG-3′; probe: 5′-FAM- ATGATGCATGATCCAGC BHQ-1 plus-3′. For amplification, the following temperature profile was used: Reverse-transcription 1 cycle at 50 °C for 5 min, denaturing and polymerase activation at 95 °C for 20 s, and amplification: 45 cycles of 95 °C for 15 s and 60 °C for 60 s. VSV-IN VR-158 (ATCC, Manassas, VA, USA) was used as an internal control for each PCR reaction and plate control for inter-run variation. Water was used for non-template negative controls. Samples were tested in triplicate and average Ct values used for analysis. Based on positive and negative controls, samples with a minimum of two RT-qPCR reactions with Ct ≤ 36.5 were considered positive for VSV-IN RNA. This cutoff value was based on our previous work with *Culicoides* midges and VSV-NJ [31,68]. Standard curves and calculation of Ct values were carried out with the 7500 Fast Dx software (Applied Biosystems; Thermo Fisher Scientific, Inc., Waltham, MA, USA). The linear regression (y = −3.3239x + 18.815) was used to determine the amount of viral genomic ssRNA per midge. Genome equivalents were calculated with the published VSV genome molecular weight [69] and the NEBioCalulator (https://nebiocalculator.neb.com/#!/ssrnaamt, access date: 13 March 2021).

### 4.4. Sequencing and Sequence Analysis

The remaining RNA from positive samples was pooled by species and submitted to the Kansas State Veterinary Diagnostic Laboratory for Illumina next generation deep sequencing of the hypervariable region of the phosphoprotein (P) gene. Full-length sequences obtained were compared to each other and to published sequences in GenBank using BLASTN 2.12.0 [30]. This included the seven sequences currently available from the 2019–2020 VSV-IN outbreak (Table S1).

### 4.5. Infection Rate Calculations

Two field infection rate metrics were calculated at each site including the MIR, a conservative estimate, and the MLE, a more robust estimate of field infection rates. MIR was calculated by dividing the total number of positive pools by the total number of insects sampled and multiplying by 1000 such that the resulting number is the number of infected insects on the landscape out of 1000 individuals. The MLE was calculated using the Excel PooledInfRate program (available through the CDC) adapted for variable pool sizes, which uses a binomial distribution and a probabilistic model to find a more likely and less conservative estimate of field infection rates [70,71].

## Figures and Tables

**Table 1:** *Culicoides* and *Simulium* individuals collected at the Franklin and Riley County sites.

Species	Franklin N	Franklin Pools Total (+)	Riley N	Riley Pools Total (+)
*Culicoides crepuscularis*	16	6 (0)	160	16 (0)
*Culicoides guttipennis*	1	1 (0)	0	0 (0)
*Culicoides haematopotus*	289	29 (0)	86	14 (0)
*Culicoides sonorensis*	1	1 (0)	148	14 (1)
*Culicoides stellifer*	120	15 (0)	266	19 (3)
*Culicoides variipennis*	0	0 (0)	266	27 (1)
*Culicoides venustus*	1	1 (0)	1	1 (0)
*Culicoides villosipennis*	1	1 (0)	0	0 (0)
*Simulium meridionale*	40	9 (3)	0	0 (0)
Total	469	63 (3)	927	91 (5)

Total number (N) is shown as well as the number of pools for each species and the total positive pools in parenthesis.

**Table 2:** Sex and parity status of *Culicoides* biting midge pools tested for VSV-IN by RT-qPCR. Parity status was not determined for nine pools of female *Simulium meridionale*.

		Female		
*Culicoides* Species	Nulliparous	Parous/Gravid	Blood Fed	Male
*C. crepuscularis*	6 (0)	13 (0)	1 (0)	2 (0)
*C. guttipennis*	0 (0)	0 (0)	0 (0)	1 (0)
*C. haematopotus*	8 (0)	21 (0)	8 (0)	6 (0)
*C. sonorensis*	2 (1)	5 (0)	3 (0)	5 (0)
*C. stellifer*	5 (0)	24 (3)	2 (0)	3 (0)
*C. variipennis*	4 (1)	11 (0)	3 (0)	9 (0)
*C. venustus*	1 (0)	1 (0)	0 (0)	0 (0)
*C. villosipennis*	0 (0)	1 (0)	0 (0)	0 (0)

Positive pools indicated in parentheses.

## Data Availability

The data presented in this study are available upon request from the corresponding authors and through the USDA, Agricultural Research Information System.

## Supplementary Materials

The following is available online at https://www.mdpi.com/article/10.3390/pathogens10091126/s1, Table S1: Vesicular stomatitis virus Indiana sequences downloaded from GenBank.
