# Supplementary material for: Detection of Vesicular Stomatitis Virus Indiana from Insects Collected during the 2020 Outbreak in Kansas, USA
Source: Pathogens. 2021 Sep 2;10(9):1126. doi: 10.3390/pathogens10091126 (PMC8471201; doi:10.3390/pathogens10091126)
Supplement: Supplementary file 1 [file pathogens-10-01126-s001.zip › pathogens-1342920-supplementary.pdf]

Table S1: Vesicular stomatitis virus Indiana sequences downloaded from GenBank

GenBank Accesion	Isolate	Isolation data
MW373779	IN0820KSE	Swabs from Kansas horses in 2020
MW373778	IN0720KSE3	Swabs from Kansas horses in 2020
MW373777	IN0720KSE2	Swabs from Kansas horses in 2020
MW373776	IN0720KSE	Swabs from Kansas horses in 2020
MT437285	IN0919COB	Swabs from Colorado cattle in 2019
MT437284	IN0919WYB1	Swabs from Wyoming cattle in 2019
MT437283	IN0919WYB2	Swabs from Wyoming cattle in 2019
